# Editorial: Molecular mechanisms of nociception

**DOI:** 10.3389/fnmol.2022.1025230

**Published:** 2022-09-06

**Authors:** Cyril Goudet, Fabien Marchand

**Affiliations:** ^1^IGF, University of Montpellier, CNRS, INSERM, Montpellier, France; ^2^Université Clermont Auvergne, Inserm U1107 Neuro-Dol, Pharmacologie Fondamentale et Clinique de la Douleur, Clermont-Ferrand, France

**Keywords:** pain, nociception, chronic pain, physiopathologic mechanism, therapeutic targets, analgesic

Nociception is the perception of noxious stimuli (chemical, thermal, or mechanical) by neurons of the nociceptive system through the peripheral and central nervous system. This is a highly complex phenomena involving different key molecules, activating different types of neurons and pathways. In addition, the nociceptive system is dysregulated in a context of chronic pain altering the process as a whole. Getting insights into nociceptive processes will lead to a better understanding of chronic pain physiopathology and will certainly lead to the discovery of new therapeutic targets.

This Research Topic covers a large spectrum of nociceptive mechanisms, from the periphery to the brain, proposing new mechanisms and/or potential new therapeutic targets.

First, we gathered articles investigating nociception through a developmental perspective such as Tröster et al. who studied the impact of axon bifurcation on sensory processing in adulthood or how transcription factors such as PRDM12 are required for nociceptor function throughout life (Kokotović et al.).

Some of these articles investigated the modulation of the nociceptive message at the spinal cord level. Leonardon et al. nicely demonstrated that NMDA receptors modulate GABAergic transmission reinforcing the role of plastic changes in synaptic inhibition which can be ultimately involved in the development and maintenance of chronic pain. Another study by Comitato et al. showed that 5-HT7 receptors also regulate excitatory-inhibitory balance in mouse spinal dorsal horn. These two studies highlight the role of the imbalance of inhibition and excitation and their mutual regulation underlying chronic pain development and/or maintenance.

Then, nociceptive signal reaches the brain where it is integrated. Here, Presto and Neugebauer observed a sexual dimorphic function of CGRP in the amygdala, indicating that CGRP1 receptors could be potential therapeutic targets for neuropathic pain relief, particularly in female. Two others studies investigated molecular changes in the locus coeruleus and the prelimbic cortex. Short-term pain seems to lead to an increase of markers of excitatory synapses in the perisomatic region of noradrenergic cells in the locus coeruleus, an effect that is lost after long-term pain, which appears to rather activate apoptosis (Bravo et al.). In the other hand, Fan et al. demonstrated that persistent hyperphosphorylation of p38MAPK in the prelimbic cortex underlies aggravated nociceptive responses in rats with chronic inflammatory pain. Overall, these studies highlight how chronic pain altered the functioning of different brain area.

A better understanding of the factors and receptors leading to chronic pain will be also clearly helpful. In the last decade, lipids have emerged as potential contributors of chronic pain. Pidoux et al. found that a single intraplantar injection of lysophosphatidyl-choline through peripheral ASIC3 activation lead to an increase of dorsal horn neuronal activity, underlining the potential of modulating lipids for chronic pain treatment.

Unfortunately, chronic pain treatments lack of efficacy and often lead to adverse effects. Here, Oehler et al. and Notartomaso et al. demonstrated the potential of botulinum toxin A1 and cinnabaric acid, respectively, to reduce chronic pain. These two studies also improved our understanding of their mechanisms of actions. Conversely, a better knowledge of the mechanism of action of efficacious analgesic could allow to target specific neuronal subpopulation and/or pathways. Thus, Ma et al. observed that the population of *Trpv1* and *Oprm1* co-expressing neurons may explain the remarkable efficacy of opioid drugs administered at the level of the DRG-spinal synapse, and that this subpopulation of *Trpv1*+ neurons is responsible for registering tissue damage. In addition, Petitjean et al. reviewed the analgesics properties of monoterpenes through their modulation of TRP channels activity and the importance and the potential of characterizing new plant extracts for the development of ethnopharmacology-based innovative treatments for chronic pain.

Finally, relevant rodent models of chronic pain inspired by patients will definitely help to improve the translational quality of preclinical studies. In this Research Topic, Xue et al. provided genetic evidence that the SCN9AR185H point mutation of Nav1.7 channel plays an important role in nociception and in pain experienced by patients with small fiber neuropathy suffering from this mutation. These findings should help to further explore pain treatments based on the Nav1.7 channel.

In conclusion, we hope that this Research Topic, which covers a large spectrum of nociceptive mechanisms, will be useful for scientists interested in understanding the physiopathology of chronic pain and the identification of potential therapeutic targets.

## Author contributions

Both authors listed have made a substantial, direct, and intellectual contribution to the work and approved it for publication.

## Conflict of interest

The authors declare that the research was conducted in the absence of any commercial or financial relationships that could be construed as a potential conflict of interest.

## Publisher's note

All claims expressed in this article are solely those of the authors and do not necessarily represent those of their affiliated organizations, or those of the publisher, the editors and the reviewers. Any product that may be evaluated in this article, or claim that may be made by its manufacturer, is not guaranteed or endorsed by the publisher.

